# Changes in HLA-B27 Transgenic Rat Fecal Microbiota Following Tofacitinib Treatment and Ileocecal Resection Surgery: Implications for Crohn’s Disease Management

**DOI:** 10.3390/ijms25042164

**Published:** 2024-02-10

**Authors:** Aurélie Blondeaux, Caroline Valibouze, Silvia Speca, Christel Rousseaux, Caroline Dubuquoy, Hélène Blanquart, Philippe Zerbib, Pierre Desreumaux, Benoît Foligné, Marie Titécat

**Affiliations:** 1 U1286—INFINITE—Institute for Translational Research in Inflammation, CHU Lille, Inserm, Univ. Lille, F-59000 Lille, France; aurelie.blondeaux59@gmail.com (A.B.); caroline_valbouze@hotmail.fr (C.V.); silvia.speca@univ-lille.fr (S.S.); philippe.zerbib@chu-lille.fr (P.Z.); pdesreumaux@hotmail.com (P.D.); marie.titecat@univ-lille.fr (M.T.); 2 Department of Hepato-Gastroenterology, Lille University Hospital, 59037 Lille, France; 3 Intestinal Biotech Development, 1 Avenue Oscar Lambret, 59045 Lille, France; crousseaux@ibd-biotech.com (C.R.); carodubu@yahoo.fr (C.D.); 4 GenoScreen, 1 Rue du Pr Calmette, 59000 Lille, France; helene.blanquart@genoscreen.fr

**Keywords:** Crohn’s disease, HLA-B27 transgenic rat model, microbiota, tofacitinib, ileocecal resection, postoperative occurrence

## Abstract

The therapeutic management of Crohn’s disease (CD), a chronic relapsing–remitting inflammatory bowel disease (IBD), is highly challenging. Surgical resection is sometimes a necessary procedure even though it is often associated with postoperative recurrences (PORs). Tofacitinib, an orally active small molecule Janus kinase inhibitor, is an anti-inflammatory drug meant to limit PORs in CD. Whereas bidirectional interactions between the gut microbiota and the relevant IBD drug are crucial, little is known about the impact of tofacitinib on the gut microbiota. The HLA-B27 transgenic rat is a good preclinical model used in IBD research, including for PORs after ileocecal resection (ICR). In the present study, we used shotgun metagenomics to first delineate the baseline composition and determinants of the fecal microbiome of HLA-B27 rats and then to evaluate the distinct impact of either tofacitinib treatment, ileocecal resection or the cumulative effect of both interventions on the gut microbiota in these HLA-B27 rats. The results confirmed that the microbiome of the HLA-B27 rats was fairly different from their wild-type littermates. We demonstrated here that oral treatment with tofacitinib does not affect the gut microbial composition of HLA-B27 rats. Of note, we showed that ICR induced an intense loss of bacterial diversity together with dramatic changes in taxa relative abundances. However, the oral treatment with tofacitinib neither modified the alpha-diversity nor exacerbated significant modifications in bacterial taxa induced by ICR. Collectively, these preclinical data are rather favorable for the use of tofacitinib in combination with ICR to address Crohn’s disease management when considering microbiota.

## 1. Introduction

Crohn’s disease (CD) is a chronic relapsing–remitting pathology with a multifaceted etiology involving genetic predisposition, environmental factors and intestinal dysbiosis combined with an aberrant immune reaction against the gut microbiota [1,2]. The role of the microbiome and its associated metabolome in the host’s immune and metabolic pathological signaling in CD is well established so far [3,4,5]. The therapeutic management of Crohn’s disease is highly challenging, as no treatments are yet curative. Indeed, many CD patients are non-responders and/or become (progressively) refractory to anti-inflammatory molecules. Of note, interactions between the intestinal microbiota and relevant IBD drugs are crucial, as treatments may directly affect taxonomic microbial profiles, whereas specific commensal microbes can conversely metabolize therapeutic molecules and strongly modify pharmacokinetics [6]. This may obviously contribute to explaining distinct individual responses and prognostic outcomes to various treatments of this complex chronic inflammatory bowel disease [7]. 

Moreover, given the high frequency of necessary surgical resections of macroscopic ileocecal or colonic lesions (30% to 50% throughout the patient’s lifetime), predominant subclinical (endoscopic) postoperative recurrences at anastomotic sites (reaching up to 70% rate) and further clinical extents also have to be taken into account to improve the efficiency of either prophylactic or therapeutic approaches. The physiopathology-sustaining postoperative recurrences of Crohn’s disease remain unknown, although interactions between the enteric and systemic immune system and the gut microbiota at mucosal sites are highly suggested [8,9]. Additionally, it is also well established that overall intestinal surgeries, including ileocecal resection and colorectal surgery, can strongly modify the gut microbiome structure and affect its functionality [10,11,12,13,14]. Collectively, gut microbiota perturbation is thus central in (i) the contribution of the clinical parameters of Crohn’s disease (initiation, maintenance and perpetration of inflammation); (ii) the postoperative recurrence of the disease, defining resilience and remission following surgical intervention; and (iii) drug-based therapeutic responses, considering bidirectional interactions between microbes and active molecules.

The HLA-B27/β_2_ microglobulin transgenic rat is a good model for mimicking inflammatory disorders broadly used in IBD research. It includes several drugs [15,16,17] and dietary interventions targeting colitis-likely diet types, such as exclusive enteral nutrition impact [18], nutrients [19,20], minerals [21], antioxidants [22,23], prebiotics [24,25,26] and probiotics [24,27,28,29]. The HLA-B27 rat model has revealed gut dysbiosis with similar intestinal microbiome traits to those seen in CD [30,31,32,33,34,35]. Recently, a reliable model of postoperative recurrence of gut inflammation was successfully modeled via ileocecal resection in HLA-B27 transgenic rats [29]. In addition, based on evidence that an abnormal JAK/STAT activation is involved in postoperative occurrence [36], a 2-week preventive treatment with tofacitinib—a potent and orally active small-molecule Janus kinase inhibitor highly selective to JAK-1 and JAK-3—is thus a favorable treatment. In the same vein, tofacitinib could prevent postoperative recurrences of Crohn’s disease via the abovementioned model of ileocecal resection in HLA-B27 transgenic rats. This was achieved through a pilot and exploratory study derived from a more extensive functional preclinical study (33 animals). In the present study, we used shotgun metagenomics to first delineate the baseline composition and determinants of the fecal microbiome of HLA-B27 rats and then, whereas little is known on the impact of tofacitinib on the gut microbiota [6,37] to evaluate the distinct impact of either a two-week tofacitinib treatment, ileocecal resection or the cumulative effect of both interventions on the gut microbiota in HLA-B27 rats.

## 2. Results

### 2.1. Bacterial Intestinal Ecology of the HLA-B27 Rat at Baseline (W9)

**Figure 1:**
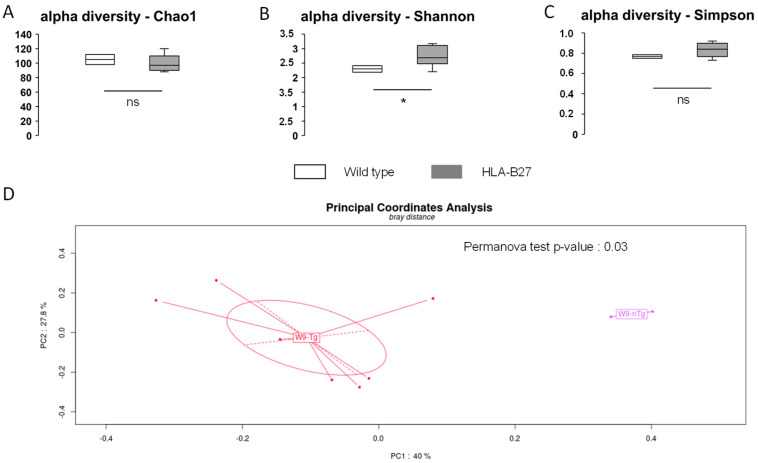
Distinct α- and β-diversity in intestinal ecology of wild-type and transgenic HLA-B27 rats. (**A**) Chao1 α-diversity index; (**B**) Shannon α-diversity index; (**C**) Simpson α-diversity index. Box and whisker plots for wild-type (white bars) and HLA-B27-Tg rats (gray bars). (**D**) Bray–Curtis distance in principal coordinate analysis as β-diversity measurement between wild-type (W9-nTg) and transgenic HLA-B27 rats (W9-Tg). Statistically significant *p*-values are marked with an asterisk. * *p* < 0.05; ns: non-significant.

**Figure 2:**
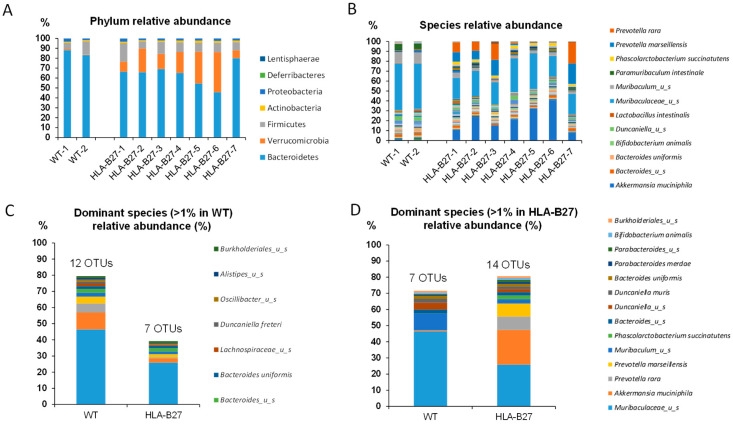
Distinct bacterial relative abundances of the fecal microbiota of wild-type and transgenic HLA-B27 rats. (**A**) Individual relative abundance at the phylum level and (**B**) at the species level. (**C**) Selective representation of the dominant species (>1% in wild-type rats) in wild-type and HLA-B27 rat groups. (**D**) Selective representation of the dominant species (>1% in HLA-B27 rats) in wild-type and HLA-B27 rat groups. Data are expressed in relative % of total phylum and species abundance, respectively, for wild-type and HLA-B27-Tg rats.

**Figure 3:**
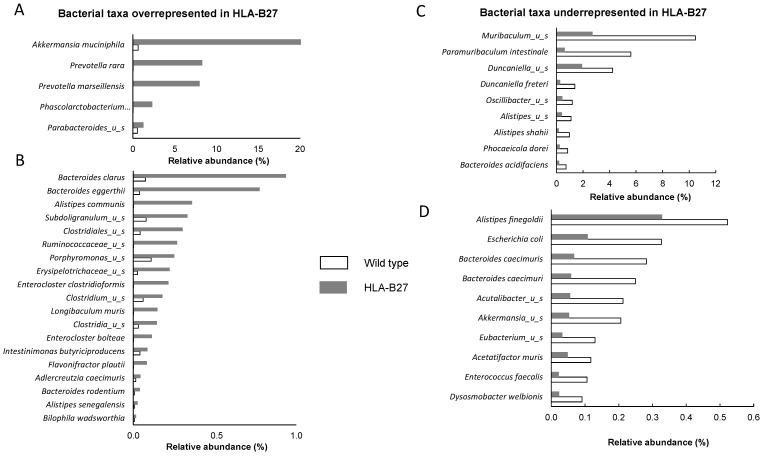
Distinct bacterial species relative abundances of the fecal microbiota of wild-type (white bars) and transgenic HLA-B27 (grey bars) rats. (**A**) Representation of bacterial taxa overrepresented in HLA-B27 rats among dominant species (>1% of relative abundance). (**B**) Representation of bacterial taxa overrepresented in HLA-B27 rats among sub-dominant species (<1% of relative abundance). (**C**) Representation of bacterial taxa underrepresented in HLA-B27 rats among dominant species (>1% of relative abundance). (**D**) Representation of bacterial taxa underrepresented in HLA-B27 rats among sub-dominant species (>1% in HLA-B27 rats).

When comparing the fecal microbiota composition of 9-week-old age-matched wild-type (not transgenic) rats (n = 2) with HLA-B27 (n = 7), the overall alpha diversity was not affected, as reported by the Chao1, Simpson and Shannon indices (respectively, Figure 1A-B-C). PCoA multivariate analysis clearly revealed significant differences between the two groups (Figure 1D) (*p*-value = 0.03). Of note, the relative abundance of bacterial species of individuals was relatively consistent within groups independent of any cage or gender effect both at the phylum and species levels (Figure 2A and B, respectively). However, as previously reported, the distribution of bacterial taxa from HLA-B27 showed substantial differences within the dominant species (>1% of relative abundance) correspondingly in wild-type and transgenic rats (Figure 2C,D). Indeed, some dominant and rarer taxa were significantly overrepresented in HLA-B27. Of note, *Akkermansia muciniphila* was drastically more present, dropping from 0.6% in wild-type rats to up to 21% (*p* < 0.05). *Prevotella rara* and *Prevotella marseillensis*, which are undetectable in wild-type rats, both reached 8% in HLA-B27 (*p* < 0.05 and *p* < 0.01). Among the rare species detected in the wild-type animals, several taxa were also strongly more abundant, with fold increases ranging from 4 to 200 times, like some *Bacteroides* spp. and *Alistipes communis* and several *Clostridiaceae*. Conversely, specific bacteria in the wild-type animals had selectively less abundance when compared with HLA-B27 (Figure 3C,D). This includes, for instance, *Miraculum* spp. (*p* < 0.05); *Duncaniella* spp. (*p* < 0.05); *Bacteroides caecimuri* (*p* < 0.05); *Enterococcus faecalis*; and *E. coli*.

### 2.2. Impact of Tofacitinib Treatment on Wild-Type and HLA-B27 Rat Microbiota

**Figure 4:**
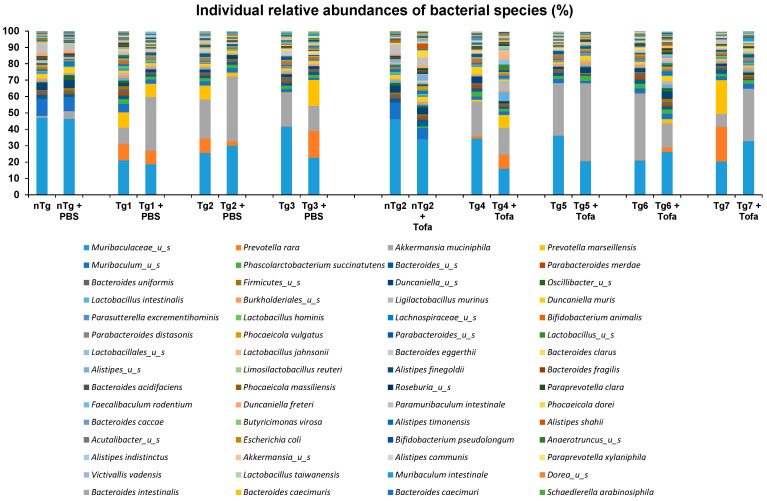
Impact of 2-week oral tofacitinib treatment on rat gut microbiota. Individual bacterial species relative abundances of the fecal microbiota of wild-type (nTg) and transgenic (Tg) HLA-B27 rats. Animals were fed with either saline (PBS) or tofacitinib (Tofa). Data are expressed in relative % of total species abundance.

**Figure 5:**
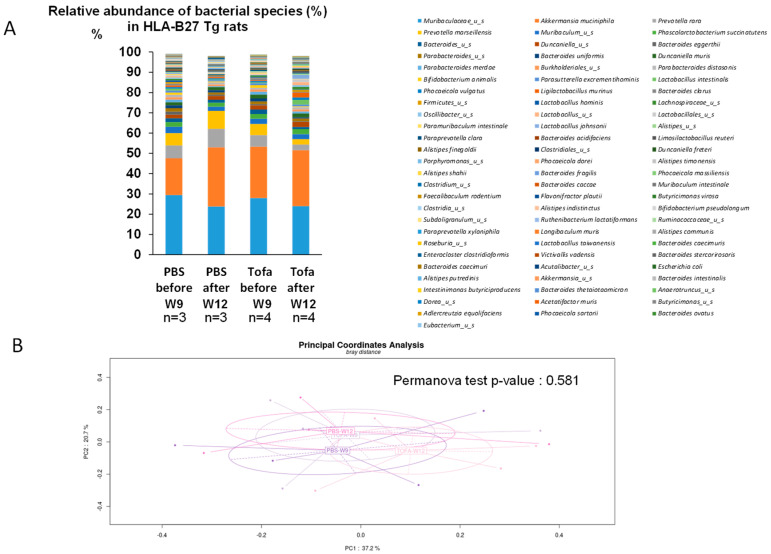
Impact of tofacitinib on the gut microbiota of HLA-B27 rats. (**A**) Bacterial species relative abundance of the fecal microbiota of wild-type HLA-B27 rats fed with either saline (PBS) or tofacitinib (Tofa) before (W9) or after (W12) a 2-week oral treatment. Data are expressed in % of total species abundance. (**B**) Bray–Curtis distance (principal coordinate analysis) as a β-diversity measurement of fecal microbiota before saline (PBS-W9) or tofacitinib (TOFA-W9) or after corresponding treatments, respectively, PBS-W 12 and TOFA-W12.

**Figure 6:**
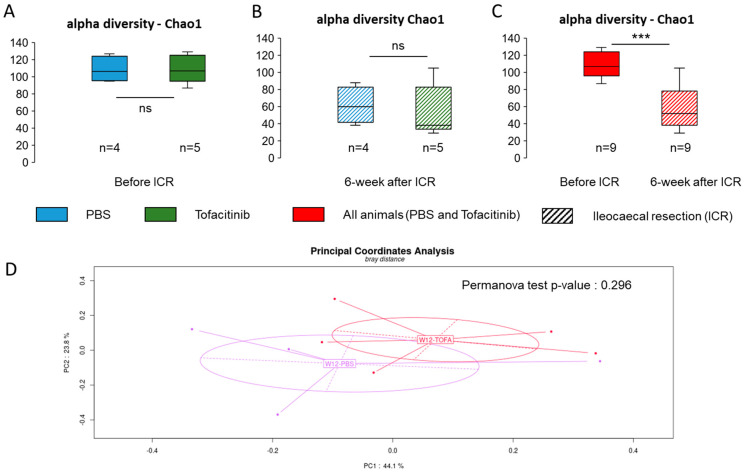
Impact of tofacitinib and ileocecal resection (ICR) on rat fecal microbiota. (**A**) Chao1 α−diversity index of 2-weeks saline- (PBS) and tofacitinib-treated rats before ICR (W9). (**B**) Chao1 α−diversity index of 9-weeks saline- (PBS) and tofacitinib-treated rats 6 weeks after ICR. (**C**) Chao1 α−diversity index of all rats 6 weeks after ICR (red bars). Box and whisker plots for PBS (blue bars) and tofacitinib (green bars) before ICR (plain bars) or after ICR (hatched bars). Statistically significant *p*-values are marked with an asterisk and written in bold. *** *p* < 0.001, ns: non-significant. (**D**) Bray–Curtis distance in the principal coordinate analysis as β-diversity measurement between saline- (W12-PBS) and tofacitinib-treated rats (W12-TOFA).

As a pilot study, the impact of a 2-week daily oral treatment with tofacitinib on microbiota composition was evaluated in five rats (four HLA-B27 and one wild type) and compared with saline (three HLA-B27 and one wild type). Paired evolutions in the nine individuals following the respective treatments only showed moderate and inconsistent changes (Figure 4). The overall impact of tofacitinib on the rat microbiome thus appears to be marginal and no more marked than the slight 3-week time shift of the commensal inhabitants (Figure 5A,B). Consequently, no significant effect of either the 2-week PBS or tofacitinib treatments on fecal microbiota alpha diversity was identified at week 12 (Figure 6A), and neither is depicted by Bray distance following PCoA multivariate analysis (*p*-value = 0.296) (Figure 6D).

### 2.3. Impact of Ileocecal Resection (ICR) on Wild-Type and HLA-B27 Rat Microbiota 

**Figure 7:**
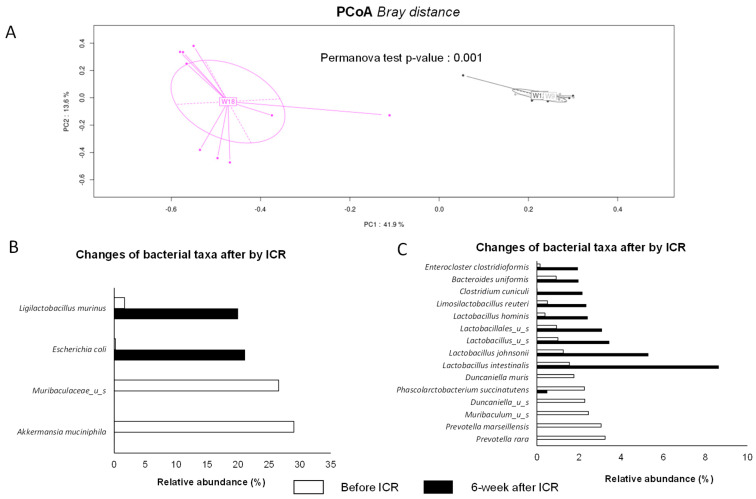
Impact of ileocecal resection (ICR) on rat fecal microbiota. (**A**) Bray–Curtis distance in principal coordinate analysis (PCoA) as β-diversity measurement before W9 and W12 and after ICR (W18). (**B**) Representation of changes in bacterial taxa before (white bars) and after ICR (black bars) for the most dominant species. (**C**) Representation of bacterial taxa overrepresented after ICR for the most dominant species. Data are expressed in relative % of total species abundance.

All animals (n = 9) were subjected to ileocecal surgery, whereas treatments (either saline or tofacinib, respectively, three HLA-B27 and one wild type and four HLA-B27 and one wild type) were maintained for 6 weeks before microbial composition achievement. The distinct treatments (saline or tofacitinib) in both wild-type and transgenic animals had no major effect, with similar respective alpha-diversity Chao1 indices among the groups (Figure 6A,B). However, ICR strongly modified the fecal bacterial taxonomy of all animals, as demonstrated by a significant reduction in Chao1 (*p* < 0.01), corresponding to a decrease of nearly 50% in OTU richness (Figure 6C). The marked changes with ICR are evidenced by significant PCoA multivariate analysis at week 18 compared with week 9 and week 12 (*p*-value = 0.001) (Figure 7A).

**Figure 8:**
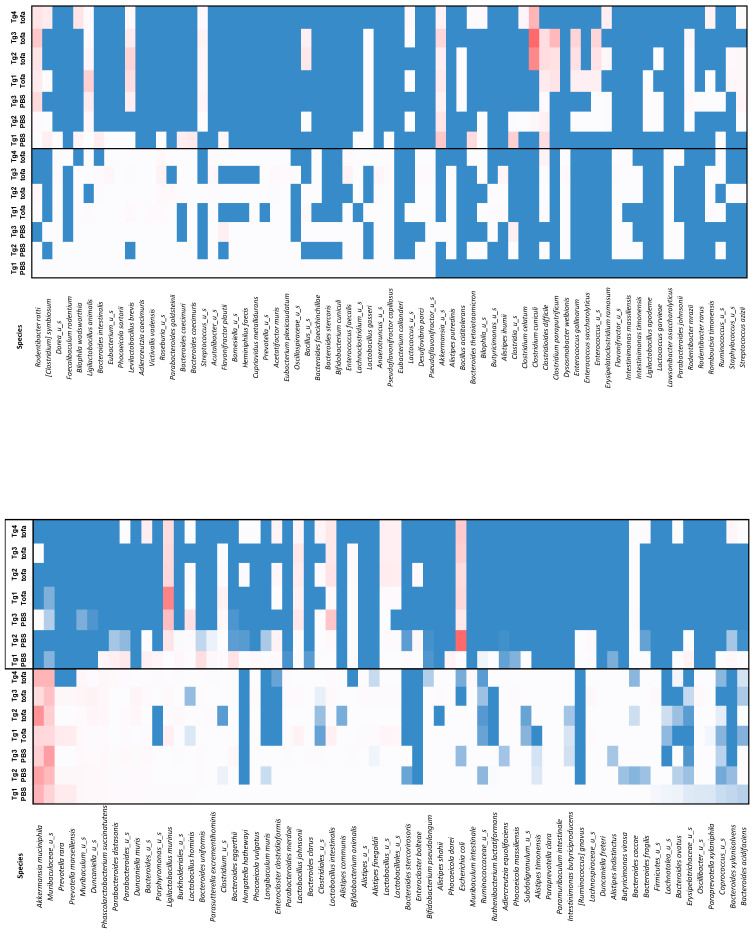
Impact of ileocecal resection (ICR) on HLA-B27-Tg rat fecal microbiota. Heatmap representation of the relative abundance of 135 bacterial taxa in individual rats before and 6 weeks after ICR. Warm (red-like) colors indicate the most abundant species, whereas cold (blue-like) colors indicate low abundance. White is unchanged abundance and light red, pink, light blue are intermediary.

**Figure 9:**
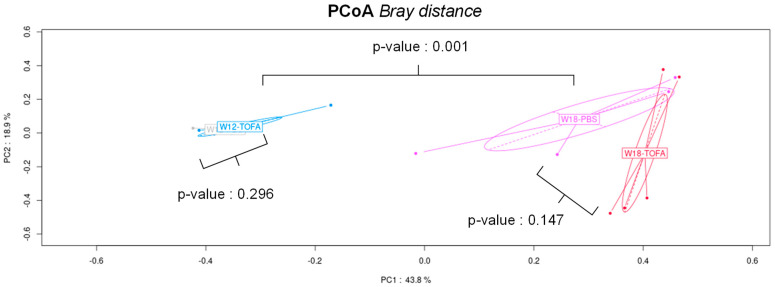
Impact of tofacitinib and ileocecal resection (ICR) on HLA-B27-Tg rat fecal microbiota. Bray–Curtis distance in principal coordinate analysis (PCoA) as β-diversity measurement for saline and tofacitinib before ICR (W9-PBS, W19-TOFA, W12-PBS and W12-TOFA) and after ICR (W18-PBS and W18-TOFA).

The most noticeable changes are represented in Figure 7. The majority of taxa, such as *Muribaculaceae* and *Akkermansia muciniphila*—representing up to 25% of the relative abundance at baseline—became undetectable after ICR, whereas rare bacterial species like *Ligilactobacillus murinus* and *Escherichia coli* each dropped to near 20% of the relative abundance 6 weeks post-surgery (Figure 7B). In addition, ICR led to a significant increase in (i) *Lactobacillus* spp. and some anaerobic taxa, such as *Bacteroides uniformis* and *Clostridium* representatives (*Enterocloster clostridioformis* and *C. cuniculi*). The genus *Duncaniella* (*D. muris* and unidentified species) and *Prevotella* spp. (*P. rara* and *P. marseillensis*), which were all at least represented by 2% of the fecal microbiota, became negligible in terms of abundance (Figure 7C). The overall drastic changes in microbial richness and composition associated with ICR are also visualized in a heatmap (Figure 8). In the same vein, our principal coordinate analysis revealed a non-significant Bray–Curtis distance between the animals between week 9 and week 12, regardless of treatments (saline versus tofacitinib), whereas a significant extended distance was demonstrated for all animals at week 18, after ICR; treatment-discriminating saline with tofacitinib had no overall effect, as seen at week 18 when combined with ICR (*p*-value 0.147) (Figure 9). However, the analysis of fecal microbiota in the experimental design focused on the most abundant bacterial species and showed that treatment with tofacitinib could recapitulate some rare but specific changes. Only a few bacterial species, such as *Akkermansia muciniphila*, *Bacteroides clarus*, *Bacteroides eggerthii* and *Phascolarctobacterium succinatutens*, were specifically influenced by tofacitinib in the context of ICR (Supplementary Scheme S1). 

## 3. Discussion

The aim of this “mostly descriptive” research study was to delineate the baseline composition and changes in the fecal microbiome of rats in the context of spontaneous inflammation and follow further treatments to target this original Crohn’s disease model. The present study (i) confirms that the microbiome of HLA-B27 rats is fairly different from their wild-type littermates and (ii) indicates that oral treatment with tofacitinib in HLA-B27 rats does not affect the gut microbial composition and (iii) neither increases the loss of bacterial diversity nor exacerbates dramatic changes in (pro-inflammatory) taxa induced by ICR. 

Indeed, although only two age-matched wild-type rats were used, we first confirmed that HLB-B27 expression in the Fisher genetic background per se clearly modified the composition of fecal microbiota compared to littermates. This was previously reported in both the Fisher and the Lewis rat genetic backgrounds, which are both associated with gut inflammation [35]. Of note, HLA-B27 transgenic rats in a third genetic background i.e., Dark Agouti, resistant to gut inflammation and without arthritis traits, showed no overall modification of intestinal microbiota. In this context, it is, however, difficult to define specific dysbiotic microbes in HLA-B27 rats, as they are highly dependent on individuals (along with time, according to disease variability); the environment; genetic backgrounds; and even the anatomical sample type and site, e.g., the cecum and colon, lumen or mucosa and fecal pellets. Gill and colleagues hypothesized the pro-inflammatory pathobiont role of *Candidatus arthromitus* (segmented filamentous bacteria, also known as SFB), which is predominant in both HLA-B27 Levis and Fisher rats but lacking in Dark Agouti rats. On the one hand, *Candidatus arthromitus* was not detected in either wild-type or HLA-B27 Fisher rats from our facility, suggesting the non-essential, non-universal role of *C. arthromitus*-promoted inflammation. It may support pluralism redundancy among deleterious bacterial species according to the environment. On the other hand, the increase in *Akkermansia muciniphila* in HLA-B27 Fisher rats we noticed here was also previously reported in spondyloarthritis models. The dual role of *Akkermansia* is intriguing, as here, the bacterium is linked to an inflammatory context, whereas it has been largely claimed elsewhere to bring benefits in metabolic issues such as obesity and diabetes in mice and even humans [38]. The intrinsic capacity of *A. muciniphila* to degrade the mucus and to disrupt the gut barrier, together with exacerbating *Salmonella*–induced inflammation [39] and food allergy [40], may prompt us to classify the bacterium as pro-inflammatory. In contrast, a large body of evidence suggests that *A. muciniphila* strains should be considered beneficial, including recommendations to address some inflammatory events via probiotic intervention [41,42]. Similarly, the *Prevotella* genus, as a saccharolytic bacterium, was at first highly suggested to act as a symbiont, providing beneficial health properties within and outside the gut [43]. In contrast, some *Prevotella* species have also been associated with chronic inflammation [44,45] and even elevated serum IL-6 [46], underlying a dual and paradoxical effect [47]. Lastly, few data are available on the pro- or anti-inflammatory status of *Duncaniella* spp., and mostly, species and, above all, strains matter [48,49]. Consequently, it is still tricky to identify colitogenic inhabitants of the HLA-B27 rat microbiota based on the abundance or rarefaction of some specific species. Indeed, we cannot rule out the presence of regulatory bacteria in limiting and counteracting damage due to inflammation, even within an inflammatory context. It was recently discovered that profiling pathobionts from rodent models and attempts to extrapolate data to human pathologies have limits [50], and several individual and environmental factors have to be considered [51]. However, even though we cannot clearly define a dysbiotic status in HLA-B27 rats, it is interesting to address the resilience of this modified commensal flora based on drugs or surgical treatments targeting inflammation. 

Here, we report that the 2-week oral tofacitinib treatment had no significant impact on either the alpha- or beta-diversity of rat fecal microbiota, in line with any substantial changes in the relative abundances of bacterial taxa. These preclinical data, although based on a small sample size, are somewhat encouraging, as the treatment per se does not induce or aggravate a possible pre-established dysbiosis. In contrast, Hablot and colleagues found a moderate influence on microbiota following a 2-week tofacitinib treatment in mice subjected to collagen-induced arthritis, also based on a small sample size. This effect was accompanied by a strong reduction in inflammatory markers and immune cells. Noticeably, those changes were more due to a reduction in pathobiont species together with an overrepresentation of beneficial phyla [37], suggesting a more healthy treatment. Interestingly, Yadav and colleagues recently showed that ileocolonic-targeted tofacitinib, in order to lower the side effect of systemic JAK-inhibitors, was highly stable in the presence of rat colonic microbiota and even more efficient than gastric release [52]. Again, it is difficult to discriminate the direct impact of tofacitinib on the microbiota from the secondary events linked to reduced inflammation. 

The most striking effect of this study is the strong impact of ileocecal resection on the fecal microbiota, defining an intense reshaping of the microbial composition and a strong drop in the alpha-diversity. This was previously documented in both wild-type and colitis-susceptible IL-10−/− mice subjected to ICR [53,54]. The resection-induced dysbiosis in our study was highly similar to that recently reported in mice [13] and could define a signature of specific taxa. Indeed, analogous changes were measured in three distinct resection models and showed reduced diversity and a dramatic decrease in *Muribaculaceae* and *Akkermansia,* two mucus-degrading species [55], which was offset by an increase in *Proteobacteria* (mostly the aerotolerant *E. coli*) and *Firmicutes*, including *Clostridiaceae*, in both limited ICR, extended ICR and even small bowel resection (SBR) experimental models. It should be noted that these procedures and, notably, those with caecum suppression, were obviously linked to intestinal insufficiency. It is well known that anatomical reconstructions lead to changes in the physiological function and composition of gut microbiota, including ICR and small bowel SBR, as well as other intestinal surgeries, such as Roux-en-Y gastric bypass procedures [56,57,58]. All may promote a loss of diversity and the deep modification of the microbiota associated with pro-inflammatory events. Luminal exposure to oxygen facilitates the depletion of anti-inflammatory obligate anaerobes and a bloom of pro-inflammatory aerotolerant organisms comprising gammaproteobacteria and modifies short-chain fatty acid production. 

Thus, perioperative gut microbial shifts have to be taken into account for therapeutic efficiency, depending on microbiota recolonization after ICR. The postsurgical re-establishment of the intestinal microbiota population and its functional consequences on the microenvironment are poorly understood [59]. Defining the anastomotic mucosal environment could be helpful in designing the timing of therapeutics when combined with ICR. This was clearly demonstrated by Madsen and collaborators using oral tributyrin treatment [55], whereas prebiotic intervention combined with ICR was, in fact, more deleterious, exacerbating the loss of diversity, as well as systemic and local inflammation [12]. Here, tofacitinib does not seem to impact ICR-induced dysbiosis and could even be favorable, possibly by preserving the abundance of lactobacilli. 

In conclusion, the present study (i) confirms that the microbiome of HLA-B27 rats is fairly different from their wild-type littermates and (ii) indicates that oral treatment with tofacitinib in HLA-B27 rats does not affect the gut microbial composition and (iii) neither increases the loss of bacterial diversity nor exacerbates dramatic changes in (pro-inflammatory) taxa induced by ICR. Collectively, these preclinical data are rather favorable for the use of tofacitinib in combination with ICR to address Crohn’s disease management in the broadest context possible. Future research directions may also be highlighted.

## 4. Materials and Methods

### 4.1. Chemicals and Reagents

Chemicals and reagents were purchased from Sigma-Aldrich Chemical (Saint-Quentin-Fallavier, France) unless otherwise stated. Ultrapure water corresponds to PURELAB Option-Q from Veolia Water (Saint-Maurice, France). Tofacitinib citrate (Pfizer, Paris, France) (Tofa) was dissolved in PBS and administered twice daily by oral gavage at the optimal concentration of 15 mg/kg/d according to previous functional studies performed in experimental murine models with colitis [60] and corresponding to daily induction doses for the treatment of ulcerative colitis in humans [61].

### 4.2. Animals

HLA-B27 transgenic (Tg) and nontransgenic (nTg) control Fisher rats (strain F344) were provided by Professor M. Breban (Cochin Institute, INSERM U1016, Paris, France). In total, 9 rats (7 Tg and 2 nTg) were maintained in a specific pathogen-free (SPF) facility at the Institut Pasteur de Lille (Lille, France) and were fed a standard diet with free access to water. Animals were kept at a constant temperature of 22 °C ± 2 °C; humidity ranged between 35% and 75%; and there was a 12 h light/dark cycle. Experiments were carried out following European directive 2016/63/UE enforced by decree n°2013-118 and authorized by regional ethics committee CEEA75 (n°CEEA 2018092818147464). The animal study protocol was also approved by the French government, MINISTERE DE L’ENSEIGNEMENT ET DE LA RECHERCHE ET DE L’INNOVATION (authorization, APAFIS n°#17092-2018092818147464; decision date, 6 April 2019), for studies involving animals.

### 4.3. Experimental Design

In total, 9 age-matched rats (7 Tg and 2 nTg, males and females) were included in the current study and randomized in i. a Tg PBS group (n = 3) and ii. An nTg PBS group (n = 1), both receiving a placebo composed of PBS, and iii. a Tg Tofa group (n = 4) and iv. An nTg Tofa group (n = 1), both receiving Tofa, as previously described [29]. Placebo (saline) or tofacitinib was administered to conscious animals starting at 10 weeks of life (W10) until W18 via intragastric gavage administration using appropriate 16G straight gavage needles. At W12, rats received an ileocecal resection (ICR) of 3 cm in length with end-to-end anastomosis. The ICR procedure was blindly performed by one operator (CD) under general anesthesia with 2% isoflurane. Briefly, after an abdominal incision, the mesenteric vessels were linked before the digestive section; then, the small intestine was cut 3 cm from the valve and 3 cm from the caecum. The anastomosis was performed with single stitches by using polypropylene surgical sutures (Prolene 6.0), whereas the parietal closure was realized on muscular and then cutaneous layers by using non-absorbable sutures made of Vicryl 2.0 and then Mersuture 1, respectively. A postoperative analgesic treatment via opioid administration was provided over three days. Fecal pellets from each animal were collected and stored at −80 °C until processed at age W9 for baseline; W12 to appraise the saline versus tofacitinib 2-week treatments; and at W18 corresponding to 6 weeks after surgery and 8 weeks of tofacitinib administration. 

### 4.4. Metagenomic Analysis

An optimized and standardized DNA extraction protocol dedicated to bacterial DNA extraction from stool samples was used (GenoScreen, Lille, France). Genomic DNA extraction was performed with the QIAamp Fast DNA Stool Mini Kit (Qiagen, Courtaboeuf, France) with an optimized protocol for the lysis step. Genomic DNA extraction from swab samples was performed with the Nucleopin Microbial DNA Mini Kit (Macherey-Nagel, Hoerdt, France) with an optimized protocol for the lysis step. After DNA extraction, the concentration was quantified with the SybrGreen Assay Kit (Life Technologies, Villebon-sur-Yvette, France). The sequencing libraries were prepared by using the Illumina DNA Prep Kit (Illumina, Evry, France). Libraries were sequenced with the Illumina NovaSeq 6000 instrument, aiming for 20 M reads per sample following a paired-end 150 bp sequencing protocol. Positive and negative controls and technical replicates were included for extraction and sequencing; all passed QC. The number of raw sequence reads per sample ranged from 25 M to 40 M. Raw paired-end reads were subjected to the Cosmos ID process. 

### 4.5. Statistical Analysis

Graphs were plotted, and statistical tests were performed using the GraphPad Prism software (version 6.0; GraphPad Software Inc., San Diego, CA, USA) or Excel (Microsoft Office, Redmond, WA, USA). In all cases, experimental groups were compared with their respective controls in a nonparametric, one-way analysis of variance (a Mann–Whitney U test), as appropriate for low-count samples. Quantitative variables were quoted as the mean ± standard deviation (SD). The threshold for statistical significance was set to *p* < 0.05. For some analytical procedures and graphical representations, we used the freely accessible application Shaman [62].

## Data Availability

The data presented in this study are available upon request from the corresponding authors.

## Supplementary Materials

The supporting information can be downloaded at https://www.mdpi.com/article/10.3390/ijms25042164/s1.

## Abbreviations

IBD	Inflammatory bowel disease
POR	Postoperative recurrence
ICR	Ileocecal resection
CD	Crohn’s disease
