# Supplementary material for: Changes in HLA-B27 Transgenic Rat Fecal Microbiota Following Tofacitinib Treatment and Ileocecal Resection Surgery: Implications for Crohn’s Disease Management
Source: Int J Mol Sci. 2024 Feb 10;25(4):2164. doi: 10.3390/ijms25042164 (PMC10889215; doi:10.3390/ijms25042164)
Supplement: Supplementary file 1 [file ijms-25-02164-s001.zip › ijms-2845814-supplementary.pdf]

Supplementary Materials: The following supporting information can be downloaded at: www.mdpi.com/xxx/s1, Figure S1: title; Table S1: title; Video S1: title.

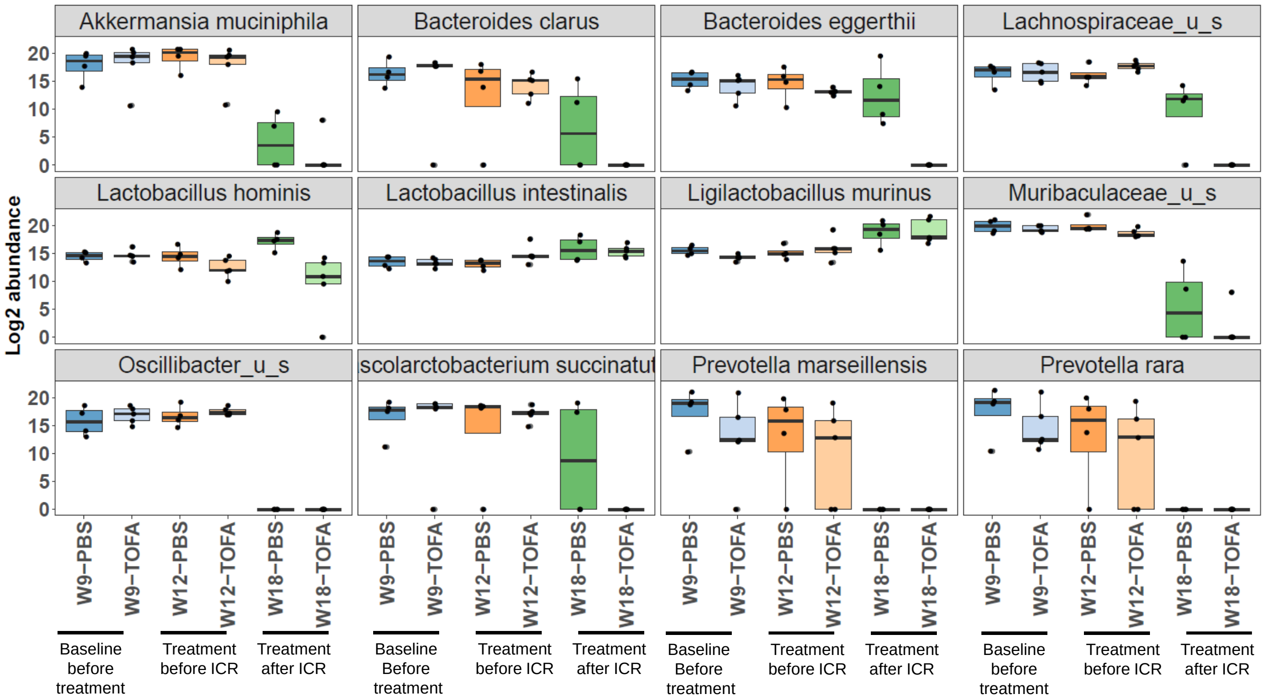

Scheme 1. Overall time-course impact of tofacitinib treatment and and ileocecal resection (ICR) on HLA-B27 Tg rat fecal microbiota. Data are expressed in Log2 abundance for the 12 most representative bacteria taxa.
